# 4381 Examination of FDA Pediatric Regulations: Inclusion of Pediatric Populations in Clinical Trials, 2016 - 2018

**DOI:** 10.1017/cts.2020.389

**Published:** 2020-07-29

**Authors:** Annie Ly, Apurva Uniyal, Terry Church

**Affiliations:** 1 University of Southern California; 2 USC School of Pharmacy

## Abstract

OBJECTIVES/GOALS: To assess whether FDA regulations aimed at the pediatric population following the Best Pharmaceuticals for Children Act (BPCA) of 2002 are effective, this study examines the inclusion of the pediatric population in recent clinical trials for drugs used by both adult and pediatric groups. METHODS/STUDY POPULATION: From the U.S. Food and Drug Administration (FDA) a list of drugs approved between 2016 and 2018 was compiled. A search of clinicaltrials.gov provided corresponding clinical trials for the approved drugs. Study information such as eligibility criteria and demographics was gathered from each trial. From studies that included both adult and pediatric populations, the percentage of pediatric and adult subjects was calculated, resulting in values expressing exclusively pediatric subjects or the pediatric subjects as part of a category that included both populations (i.e. 18 years old). RESULTS/ANTICIPATED RESULTS: Between 2016 and 2018, 26 drugs were approved under the BPCA. From an assessment of 220 total studies, a lack of standardization is evident in terms of which ages constitute a particular pediatric sub-population even though guidelines for these sub-populations already exist under the BPCA. This lack of standardization resulted in the separate examination of each drug for pediatric inclusion. For the majority of the trials evaluated, 1% of the pediatric population was represented in trials that were open to both adult and pediatric populations. DISCUSSION/SIGNIFICANCE OF IMPACT: There is a need for more effective regulations and incentives for the pharmaceutical industry to standardize data presentation and better incorporate the pediatric population in clinical trials, especially for drugs targeted for this group.

